# Protective Effect of Blood Cora Polysaccharides on H9c2 Rat Heart Cells Injury Induced by Oxidative Stress by Activating Nrf2/HO-1 Signal Pathway

**DOI:** 10.3389/fnut.2021.632161

**Published:** 2021-03-02

**Authors:** Yong Jiang, Wei Zhou, Xin Zhang, Ying Wang, Dingyi Yang, Shujie Li

**Affiliations:** ^1^Laboratory for Biorheological Science and Technology of Ministry of Education (Chongqing University), Chongqing University Cancer Hospital & Chongqing Cancer Institute & Chongqing Cancer Hospital, Chongqing, China; ^2^Chongqing Key Laboratory of Translational Research for Cancer Metastasis and Individualized Treatment, Chongqing University Cancer Hospital & Chongqing Cancer Institute & Chongqing Cancer Hospital, Chongqing, China

**Keywords:** blood cora, polysaccharide, expression, H9c2 rat heart cells, Nrf2/HO-1 signal pathway

## Abstract

The protective effect of blood cora polysaccharides (BCP) on H9c2 rat heart cells under oxidative stress was explored with the use of a H9c2 cell oxidative stress model. The ability of BCP to scavenge 2,2'-azino-bis(3-ethylbenzothiazoline-6-sulfonic acid) (ABTS), 1,1-diphenyl-2-picrylhydrazyl (DPPH), and hydroxyl radicals and its reducing power were measured *in vitro*, indicating a more powerful antioxidant effect of BCP compared to a similar concentration of vitamin C. The cellular metabolic activity was tested through the MTT [3-(4,5-dimethyl-2-thiazolyl)-2,5-diphenyl-2-H-tetrazolium bromide] assay. Additionally, the relevant oxidation indicator level within the cell supernatant and cells was tested with reagent kits, and mRNA and protein expression levels in the cells were tested through quantitative polymerase chain reaction (qPCR) and western blot. The chemical composition of BCP was determined through high performance liquid chromatography (HPLC). The results show that compared with the normal group, the model group's cell survival rate (28.75 ± 2.56%) decreased, lactate dehydrogenase (LDH) leakage and the malondialdehyde (MDA) content increased, and superoxide dismutase (SOD), catalase (CAT), and glutathione (GSH) levels decreased. The results of qPCR and western blot show that compared with the normal group, the model group's Bcl-2 associated X protein (Bax), caspase-3, nuclear factor erythroid-2 related factor 2 (Nrf2), heme oxygenase-1 (HO-1) expression, NAD(P)H:quinoneoxidoreductase 1 (NQO1), and cytochrome *c* (Cyt C) decreased, and B-cell lymphoma-2 (Bcl-2) expression was increased, with significant statistical differences. Compared with the model group, the cell survival rate for each BCP-treated group increased, the LDH leakage decreased, the SOD, CAT, and GSH levels in the cells increased, the MDA content decreased, the Bax, caspase-3, Nrf2, HO-1, NQO1, and Cyt C expression was weakened, and the Bcl-2 expression was strengthened. BCP inhibited the reduction of mitochondrial membrane potential caused by H_2_O_2_ treatment. According to the component analysis, BCP mainly consist of mannitol, ribose, glucosum anhydricum, galactose, and xylose. It was observed that the Nrf2/HO-1 signaling pathway can be activated, regulated, and controlled by functional BCP to protect H9c2 cells injured by oxidative stress.

## Introduction

Blood cora is a type of common coral grass (*Thesium chinense* Turcz.) that is black-brown in color with large branches, and mainly grows in the pollution-free waters of the south China Sea. It has strict environmental requirements for its growth, such as water temperature between 25 and 32°C. Blood cora grass grows alongside pollution-free coral reefs, and it can remove the salts from sea water between flood tide and ebb tide to concentrate minerals from the sea water and store them (1). The plant contains calcium, iron, magnesium, potassium, phosphorus, and other minerals needed by the human body, with more iron than that of the pork liver, and more calcium than that of small dried fish, as well as containing beneficial amounts of natural plant collagen, cellulose, polysaccharides, vitamins, and enzymes (2).

Polysaccharides, formed by condensation and water loss of multiple monosaccharide molecules, are carbohydrates with a complex and considerable structure (3). Polysaccharide compounds have the functions of immune adjustment, anti-virus, anti-cancer, blood sugar level regulation, antiphlogosis, and analgesia (4–6). Polysaccharide compounds also play a role in anti-aging and facial beautification through their fine antioxygenation capabilities (7, 8). The monosaccharide compounds comprising the polysaccharides generally are obtained by hydrolysis, so that the compositions of polysaccharide compounds to be determined can be accurately analyzed (9). By identifying the hydrolysed monosaccharides, the bioactive action of the polysaccharide compounds, consisting of monosaccharides, can be more accurately analyzed (10).

Oxidative stress refers to a state of imbalance between oxidation and antioxidation *in vivo* where oxidation is favored, resulting in inflammatory infiltration of neutrophile granulocytes, an increase in protease secretion, and generation of various oxidation intermediate products. There is a negative effect from oxidative stress generated by free radicals *in vivo*, and it is considered to be an important factor in the rise of caducity and disease (11). Studies show that the hazards resulting from oxidative stress can be mitigated through antioxidants. The balance between oxidants and antioxidants is disrupted by oxidative stress, which results in cell injury (12).

Oxidative stress occurs in pulmonary fibrosis, epilepsy, hypertension, atherosclerosis, Parkinson's disease, and sudden death (13–15). The generation of excessive oxygen radicals has been found in cardiac muscle tissues under many pathological conditions. Oxygen radicals produced as a result of oxidative stress will lead to myocardial damage in many cases, and thus, myocardial cell apoptosis or necrosis can be caused by oxidative stress through multiple mechanisms (16). Nrf2 is a regulatory factor involved in oxidative stress that can regulate the encoding antioxidant protein expression and inhibit the oxidative stress response. The HO-1 gene is the dependent gene of Nrf2, and its product is a type of strong antioxidant (17).

In the current study, the polysaccharide compounds contained in blood cora were analyzed, and rat heart cells were used to establish a model of myocardial cell injury and bring about oxidative stress *in vitro* in order to observe the interventional effect of blood cora polysaccharides (BCP) on cell oxidative stress, combined with the analysis of the polysaccharides and determination of their composition. We explored the action mechanism of myocardium protection implemented by inhibiting oxidative stress through the activation of the Nrf2/HO-1 signal by BCP, with the purpose of accumulating a theoretical foundation so that additional applications for this plant can be implemented.

## Materials and Methods

### Extraction of Blood Cora Polysaccharides

A solution was made of 100 g dried sample of blood cora (Yirui Xiangyun Food Franchise Store, Qingdao, Shandong, China) and 200 mL of deionized water, and extraction was conducted in a water bath at 95°C for 40 min and under ultrasound for 20 min. The obtained solution was filtered, and 200 mL water was added to the remaining filter residue. The extraction steps were repeated once more, for a total of 4 times.

After simple filtration with gauze, the 4th filtrate was poured into a round-bottom flask that was placed in a rotary evaporator for evaporation and concentration until 100 mL of filtrate remained. Then, 400 mL of absolute ethyl alcohol was added to the solution, and it was stored at 4°C overnight. The solution was transferred to a centrifuge tube, and centrifugation was conducted at 40,000 rpm for 10 min. The supernatant was suctioned to recycle the ethyl alcohol after the rotary evaporator treatment. A small quantity of hot water at 90°C was added to dissolve the sediment, absolute ethyl alcohol was added to it until the alcohol content reached 80%, and then, the solution was stored at 4°C overnight. Centrifugation was conducted at 40,000 rpm for 10 min, and the supernatant was suctioned to continue to recycle the ethyl alcohol. The solution in the lower layer was freeze-dried until it reached a solid state for obtaining coarse polysaccharides. Sufficient hot water was added to dissolve the coarse polysaccharides, and proteins were removed through the Sevag method (chloroform: n-butyl alcohol = 4:1, v/v), 4 times in total.

Next, 4 g activated charcoal granules (ACG) was added in the solution. After decolorating for 10 min, suction filtration was conducted. Absolute ethyl alcohol was added to the filtrate until the alcohol content reached 80%, and the solution was then stored at 4°C overnight (18). The supernatant was suctioned to recycle the ethyl alcohol, and the precipitated polysaccharides and sediment were freeze-dried for 12 h. Then, the sample was placed in a vacuum-drying oven at 60°C to dry. The prepared fine BCP was transferred to a beaker for later use.

### Preparation of the Liquid Sample (Derivatization)

For the preparation of the BCP solution, 20 mg of fine BCP powder was precisely weighed and transferred to a 10-mL test tube with a stopper. Then, 2 mL of H_2_SO_4_ solution at 2 mol/L was added for hydrolysis, and the tube was placed in boiling water in a water bath for 8 h. A NaOH solution at 4 mol/L was used to adjust the pH value of the hydrolyzed sample solution to neutral. Deionized water was added to reach a constant volume of 5 mL, and centrifugation was conducted at 5,000 rpm for 10 min. The supernatant was removed to obtain the BCP solution, which was stored for later use (19).

For standardized product solution preparation, small amounts of ribose, galactose, xylose, anhydrous glucose, mannitol, and fucose were each weighed in six 2-mL centrifuge tubes, and an appropriate amount of deionized water was added to each of these tubes to prepare 6 types of monosaccharide standardized solutions, each with a concentration of 2 mol/mL. A mixed solution of the 6 monosaccharide standardized product solutions was also made by uniformly mixing small equivalent amounts of each monosaccharide.

For standardized product solution derivatization, 100 μL standardized product solution was placed in a centrifuge tube, 100 μL PMP-methanol solution at 0.5 mol/L and 100 μL NaOH solution at 0.3 mol/L were added, and the tube was vortexed for 1 min to uniformly mix the solution. The tube was placed in a water bath at 70°C for 30 min for the derivatization reaction. Then, the tube was removed, cooled until it reached room temperature, and 100 μL HCl solution at 0.3 mol/L was added to adjust the solution pH value to neutral. Next, 4 mL chloroform was added to extract the standardized product solution, and the supernatant was suctioned and filtered with a 0.22-μm microporous membrane. For BCP solution derivatization, the BCP solution was treated according to the derivatization steps described above (2).

### HPLC Analysis of BCP Derivatives

The mobile phase consisted of solutions A and B: phase A consisted of methanol solution, phase B consisted of ammonium acetate buffered solution (0.05 mol/L, pH 5.5), and phase C consisted of acetonitrile solution. The sample injection amount was 10 μL, the flow rate was 1.0 mL/min, the wavelength was 250 nm, the column temperature was 30.0°C, and a Diamonsil C18 chromatographic column was used (5 μm, 4.6 × 250 mm) (Ultimate 3000, Thermo Fisher Scientific, Inc., Waltham, MA, USA). The gradient elution program is shown in Table 1.

**Table 1:** Mobile phase gradient elution procedure.

**Time (min)**	**V (Ammonium acetate, %)**	**V (Acetonitrile, %)**
0	0	100
10	18	82
25	25	75
30	30	70

### Detection of DPPH Free Radical Scavenging Ability

According to Table 2, samples and reagents of different concentrations were added for reaction in the dark for 30 min. Absorbance of the final reaction solution was measured at a wavelength of 517 nm, using vitamin C (Vc, Shanghai Yuanye Biotechnology Co., Ltd., Shanghai, China) as the positive control (20). The capability was determined using this formula: DPPH (%) = [A_3_-(A_1_-A_2_)]/A_3_ × 100.

**Table 2:** The method of DPPH assay.

**No**.	**Experiment reaction system**
A_1_	3.9 mL DPPH reaction reagent + 100 μL sample solution
A_2_	3.9 mL anhydrous ethanol + 100 μL sample solution
A_3_	3.9 mL DPPH reaction reagent + 100 μL 80% methanol

### Detection of ABTS Free Radical Scavenging Ability

According to Table 3, samples and reagents of different concentrations were added, mixed, and reacted in the dark for 6 min. Absorbance of the final reaction solution was measured at a wavelength of 734 nm, using Vc as the positive control (20). The capability was determined using this formula: ABTS (%) = [A_3_-(A_1_-A_2_)]/A_3_ × 100.

**Table 3:** The method of ABTS assay.

**No**.	**Experiment reaction system**
A_1_	5 mL ABTS reaction reagent + 200 μL sample solution
A_2_	5 mL anhydrous ethanol + 200 μL sample solution
A_3_	5 mL ABTS reaction reagent + 200 μL 80% methanol

### Detection of Hydroxyl Radical Scavenging Ability

Using ferric-reducing/antioxidant power (15), the Vc standard solution and sample solutions of different concentrations were added to 2.5 mL of phosphate-buffered saline (PBS) buffer and 2.5 mL 1% K_3_Fe(CN)_6_, mixed well, and reacted in a water bath at 50°C for 20 min. After rapidly cooling, 2.0 mL of trichloroacetic acid at 10% (v/v) was added to stop the reaction. The 2.5 mL solution was mixed with 2.5 mL of distilled water and 0.5 mL of 0.1% FeCl_3_, and maintained at room temperature for 10 min. In the control group, the Vc standard solution was replaced by 2.5 mL of methanol. The absorbance was measured at 700 nm. Using Vc as a positive control, the higher the absorbance, the stronger the ability to scavenge hydroxyl radicals.

### Cell Line

H9c2 (Cell Bank of the Chinese Academy of Sciences, Shanghai, China) cells were inoculated in high glucose Dulbecco's modified Eagle's medium (DMEM) (Solarbio Life Sciences, Beijing, China) containing 10% fetal calf serum. The cells were then placed in a thermostatic incubator containing 5% CO_2_ at 37°C for cultivation, and passage was performed every 2–3 days.

### Experimental Grouping

Cells at logarithmic phase were randomly divided into 6 groups: (i) normal group, which received no treatment; (ii) model group, where cells were cultured for 4 h with 100 μmol/L H_2_O_2_; for the (iii) low BCP (BCPL), (iv) medium BCP (BCPM), and (v) high BCP (BCPH) treating groups, DMEM complete culture solutions (without serum) were used, each with BCP concentrations of 25 μmol/L, 50 μmol/L, and 100 μmol/L, respectively; and for the (vi) vitamin C group, a vitamin C concentration of 100 μmol/L was used, where cells were treated for 12 h, and then, H_2_O_2_ with a final concentration of 100 μmol/L was added, and they were grown for 4 h.

### Using the MTT Assay to Test for Cell Viability

Each group of cells was inoculated in 96-well culture plates, with 1 × 10^5^ cells inoculated in each well. After completion of inoculation, the plates were placed at 37°C for 12 h. After full adherence of cells, different groups of cells were treated according to the above experimental methods. Then, 20 μL MTT solution (Solarbio Life Sciences) with a concentration of 5 g/L was added to the plates, which were warmed at 37°C for 4 h. After that, the supernatant was removed, and 150 μL DMSO was added to each well. After shaking the 96-well plates for 10 min, a microplate reader was utilized to determine the OD value at the wavelength of 490 nm. The cell survival rate was calculated by taking the average value of each group: cell survival rate (%) = (OD value of experimental group/OD value of normal group) × 100 (21).

### Measuring the Amount of Lactate Dehydrogenase (LDH) in Cell Culture Fluid

The cell cultivation supernatants from each group were removed, and the amount of LDH in the cell supernatants of each group was determined with a fully automated microplate reader according to the instructions for the LDH reagent kit (microplate method kit, OD_450_ 0.05–0.50, Nanjing Jiancheng Bioengineering Institute, Nanjing, Jiangsu, China).

### Measuring Superoxide Dismutase (SOD), Malondialdehyde (MDA), Glutathione (GSH), and Catalase (CAT) Levels in Myocardial Cells

The cell culture fluids from each group were removed, and 2 mL PBS was added to each well of the 6-well plates to wash the cells 2 to 3 times. An ultrasonic cell disruptor was used to disrupt cells in an ice-water bath, with 1 ultrasound burst every 3–5 s for 4 intervals. Then, centrifugation was conducted at 1,500 rpm and 4°C for 4 min, and the supernatant was removed. Finally, the SOD (WST-1 method kit, OD_550_ 0.15–0.55), MDA (TBA method kit, OD_532_ 0.13–0.65), GSH (spectrophotometric method kit, OD_420_ 0.10–0.50), and CAT (visible light method kit, OD_405_ 0.10–0.50) levels in the cell lysates of each group were measured with a fully automated microplate reader according to the manufacturer's instructions for the different reagent kits (Nanjing Jiancheng Bioengineering Institute).

### qPCR Assay

The cell nuclei were extracted using a nuclear extraction kit (Solarbio Life Sciences), and the cells (or extracted nuclei) were pulverized with a homogenizer after treating cells according to the experimental grouping. The total RNA from the cells was extracted with TRIzol™ reagent (Solarbio Life Sciences), and diluted to 1 μg/μL. Then, 1 μL of the diluted RNA solution was removed, and a reverse transcription kit was used to obtain a cDNA template. Next, 1 μL cDNA template was mixed with 10 μL SYBR Green PCR Master Mix (Invitrogen, Carlsbad, CA, USA), 1 μL upstream and downstream primers (Table 4, Thermo Fisher Scientific), and 7 μL of sterile distilled water. The mixture was reacted at 95°C for 60 s, and 40 cycles were carried out at 95°C for 15 s, 55°C for 30 s, and 72°C for 35 s. Lastly, the mixture was reacted and tested at 95°C for 30 s, and 55°C for 35 s (StepOnePlus Real-Time PCR System, Thermo Fisher Scientific), and the 2^−ΔΔCt^ method was applied to calculate the gene relative expression, using GAPDH as an internal reference (22).

**Table 4:** Primer sequences of RT-qPCR assay.

**Gene name**	**Sequence**
Bcl-2	Forward: 5′-ATGTGTGTGGAGAGCGTCAACC-3′
	Reverse: 5′-CAGAGACAGCCAGGAGAAATCAA-3′
Bax	Forward: 5′-AGACAGGGGCCTTTTTGCTAC-3′
	Reverse: 5′-AATTCGCCGGAGACACTCG-3′
Casepase-3	Forward: 5′-CATGGAAGCGAATCAATGGACT-3′
	Reverse: 5′-CTGTACCAGACCGAGATGTCA-3′
HO-1	Forward: 5′- GATAGAGCGCAACAAGCAGAA-3′
	Reverse: 5′- CAGTGAGGCCCATACCAGAAG-3′
Nrf2	Forward: 5′- TAGATGACCATGAGTCGCTTGC-3′
	Reverse: 5′- GCCAAACTTGCTCCATGTCC-3′
NQO1	Forward: 5′-AGGATGGGAGGTACTCGAATC-3′
	Reverse: 5′-TGCTAGAGATGACTCGGAAGG-3′
GAPDH	Forward: 5′-AGGTCGGTGTGAACGGATTTG-3′
	Reverse: 5′-GGGGTCGTTGATGGCAACA-3′

### Western Blot

The cells were treated according to their experimental grouping, and were washed 2–3 times with PBS buffer solution. The cells were collected, lysed at 4°C for 20 min, centrifuged, and then, the cell supernatant was collected after centrifugation. The total protein was determined using a bicinchoninic acid (BCA) protein determination kit (Easy Bio, Beijing, China). The supernatant from the cell lysate was electrophoresed in a sodium dodecyl sulfate-polyacrylamide gel electrophoresis (SDS-PAGE) polyacrylamide gel (Thermo Fisher Scientific). After completion, the protein was transferred to a polyvinylidene difluoride (PVDF) membrane. The membrane was soaked in buffer, to which primary antibodies were added, and incubated at 4°C overnight. The membrane was then washed, and secondary antibodies in buffer were added to the membrane, which was incubated at room temperature for 1 h. The membrane was washed with buffer, and target protein expression was tested with a chemiluminiscence kit (Thermo Fisher Scientific) (22).

### Mitochondrial Membrane Potential Was Measured by the JC-1 Method

After the cells were treated according to the above experimental grouping method for 12 h, the mitochondrial membrane potential was detected by the JC-1 method (23) according to the kit instructions (Solarbio, Beijing, China), and the results are expressed by fluorescence intensity.

### Statistical Processing

The data were analyzed with SPSS 23 statistics software, which performed comparisons between groups by one-way variance. *P* < 0.05 was considered statistically significant.

## Results

### Analysis of Monosaccharide Components of BCP

**Figure 1:**
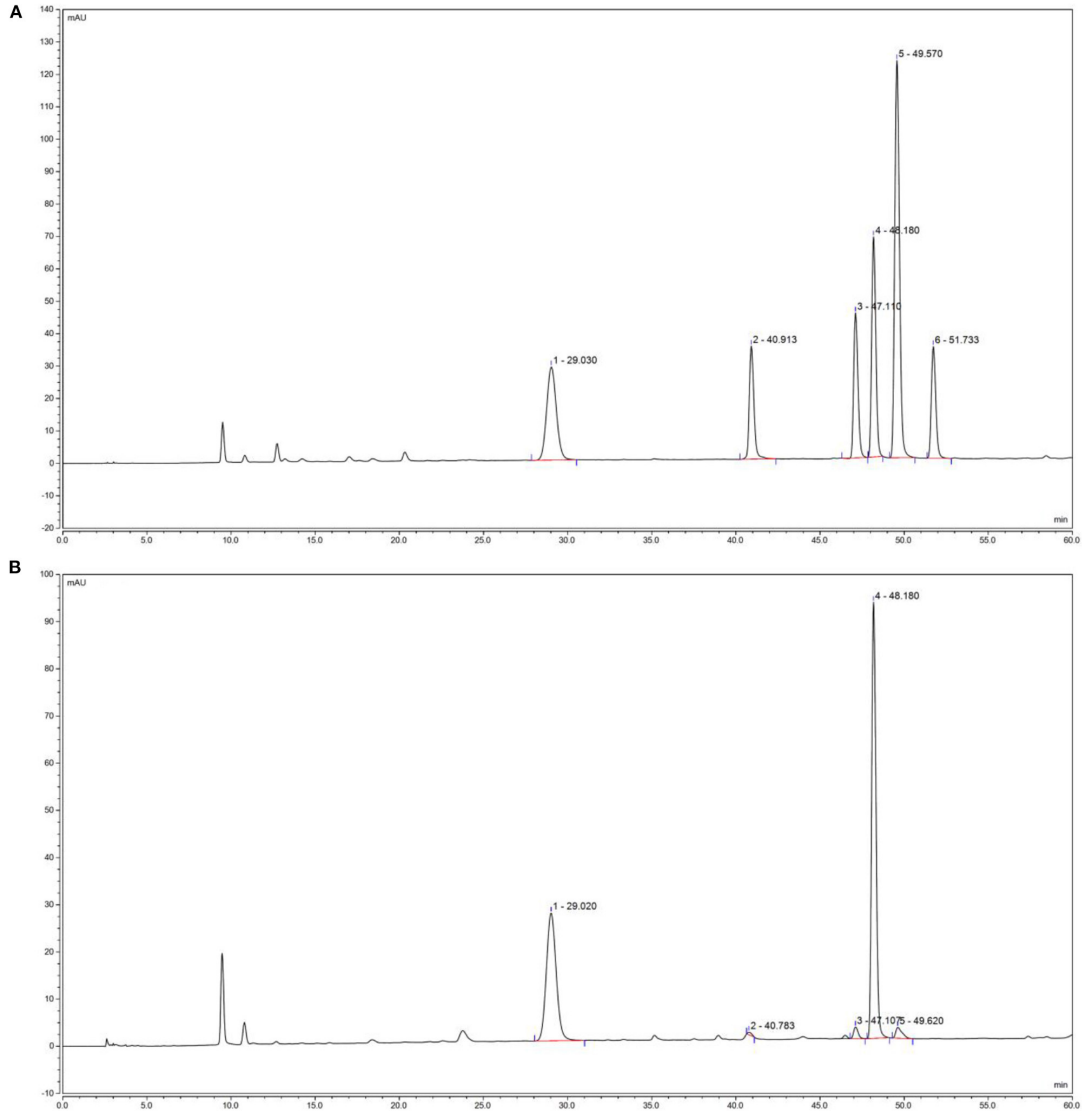
Polysaccharides constituents of blood cora. **(A)** Standard chromatograms; **(B)** blood cora polysaccharides chromatograms. 1: mannitol; 2: ribose; 3: anhydrous glucose; 4: galactose; 5: xylose.

Five derivative BCP solutions were tested, in which samples were injected every 2 h according to the chromatographic conditions specified in Section HPLC analysis of BCP derivatives. The chromatographic peak areas of the 5 samples were recorded, and a relative standard deviation (RSD) of 0.89% (*n* = 5) was obtained, which indicates the satisfactory repeatability of the determination method and that it was suitable for this experiment. The prepared mixed derivative of the standardized product solution and the BCP solution were injected according to the chromatographic conditions, and the chromatograms were recorded. Figure 1 shows that the monosaccharide components of the standardized mixed product derivative and the BCP derivative were properly separated under chromatographic conditions. A derived impurity peak appeared under 28 min that was properly separated from each peak of post monosaccharide derivatization and had no effect on the component analysis of the monosaccharide.

Compared with the high-performance liquid chromatograms of the BCP derivative and the standardized mixed product derivative, the results showed that the BCP sample contains no fucose, and its major components were mannitol, ribose, anhydrous glucose, galactose, and xylose. The derived standardized mixed product solution was injected according to the gradient order, with the sample sizes of 20 μL, 18 μL, 16 μL, 14 μL, 12 μL, and 10 μL, respectively. The peak areas of monosaccharide components were recorded at different sample sizes. Taking the sample size as the horizontal axis (x coordinate) and peak area as the vertical axis (y coordinate), canonical curve charts for the 5 monosaccharides were established in order to calculate their linear regression equations and correlation coefficients, and the amount of the monosaccharide per gram of sample was calculated with linear regression equations of 5 monosaccharides and the peak area of each monosaccharide in each sample (Table 5). The results showed that the linearly dependent coefficient *R*^2^ for the 5 monosaccharides were all > 0.99, indicating that there was a good linear correlation between the peak area and the sample size. Quantitation of each monosaccharide in 1 g blood cora polysaccharide sample was obtained through calculation, which revealed that galactose and glucosum anhydricum were present in the highest and lowest amounts in BCP, respectively.

**Table 5:** Standard curve equation and content of monosaccharide.

**Monosaccharide**	**Linear regression equation**	**Correlation coefficient *R*^**2**^**	**Content (mg/g)**
Mannitol	*y* = 1.4025x + 2.1146	*R*^2^ = 0.9943	286.18 ± 0.21
Ribose	*y* = 0.7432x + 1.9926	*R*^2^ = 0.9950	27.06 ± 0.12
Anhydrous glucose	*y* = 1.0789x + 0.1905	*R*^2^ = 0.9996	10.66 ± 0.08
Galactose	*y* = 1.5626x + 0.5028	*R*^2^ = 0.9994	446.08 ± 0.45
Xylose	*y* = 3.1653x + 2.1442	*R*^2^ = 0.9981	54.53 ± 0.31

### Ability of BCP to Scavenge DPPH

**Figure 2:**
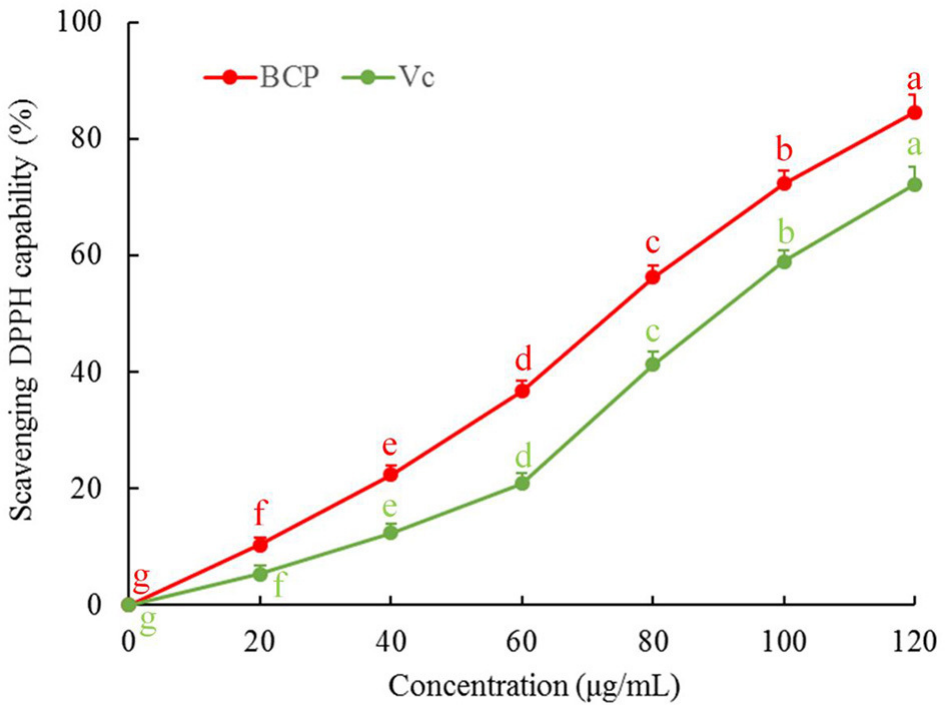
DPPH free radical scavenging capacity of blood cora polysaccharides. ^a−*g*^Mean values with different letters over the bar are significantly different (*P* < 0.05) according to Tukey's honestly significant difference.

Figure 2 illustrates that there was a dose-response relationship with regard to the ability of BCP to scavenge DPPH. In the concentration range of 0–100 μg/mL, the capability to scavenge DPPH radicals was stronger with increased concentration, and the clearance rate of DPPH in the Vc-positive control group was lower than that of BCP.

### Ability of BCP to Scavenge ABTS

**Figure 3:**
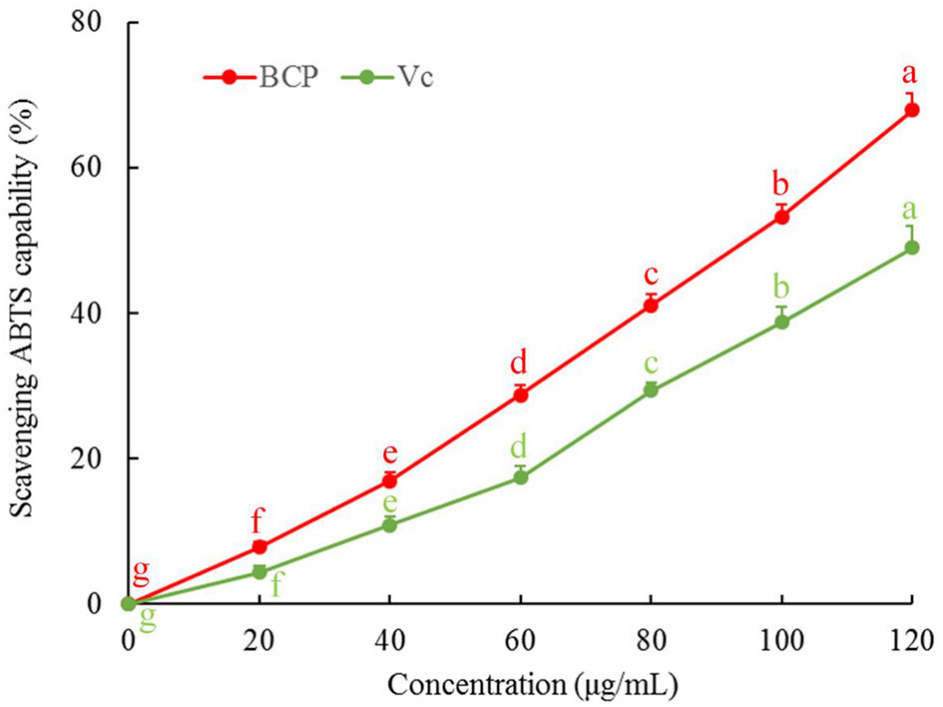
ABTS free radical scavenging capacity of blood cora polysaccharides. ^a−*g*^Mean values with different letters over the bar are significantly different (*P* < 0.05) according to Tukey's honestly significant difference.

The results showed that the ABTS clearance rate gradually increased with sample concentration. As illustrated in Figure 3, the capability of BCP to scavenge ABTS free radicals was positively correlated with the concentration. Additionally, the clearance rate of BCP to ABTS was higher than that of Vc at the same concentration.

### Ability of BCP to Scavenge Hydroxyl Radical

**Figure 4:**
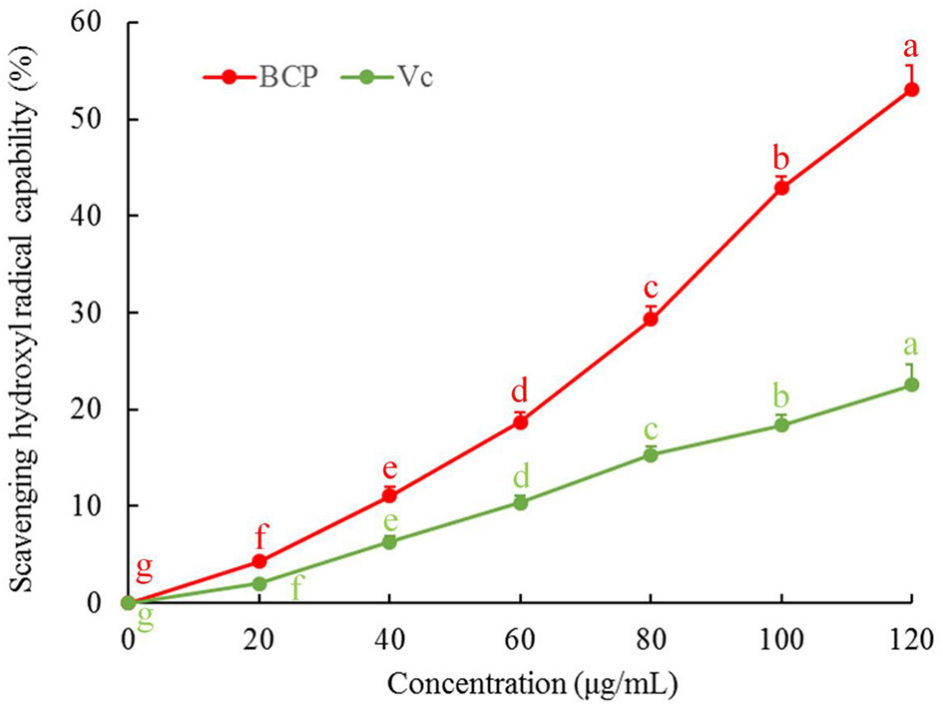
Hydroxyl radical scavenging capacity of blood cora polysaccharides. ^a−g^Mean values with different letters over the bar are significantly different (*P* < 0.05) according to Tukey's honestly significant difference.

Figure 4 illustrates that the reducing power of BCP increased with the sample concentration. When the concentration reached 40 μg/mL, the increasing amplitude of reducing power decreased, but the overall reducing power was greater than that of Vc.

### Effect of BCP on the Survival Rate of H9c2 Cells With OS Injury

**Figure 5:**
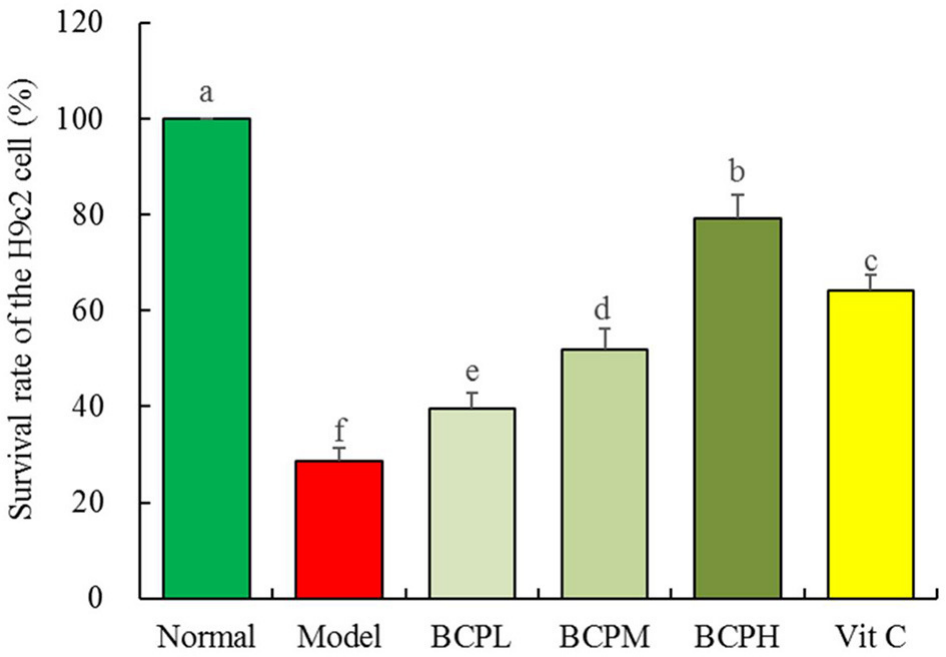
Effect of BCP on survival rate of the H9c2 cell with OS injury. ^a−*f*^ Mean values with different letters over the bar are significantly different (*P* < 0.05) according to Tukey's honestly significant difference.

The survival rate of the myocardial cells in the model group was lower than that in the normal group, and the difference was statistically significant (Figure 5, *P* < 0.05). After pretreatment with BCP at low, medium, and high concentrations, the survival rate of myocardial cells was higher than that of the model group, and the difference was statistically significant (*P* < 0.05). Dose dependence was also noted, and the survival rate of the BCPH group was higher than that in the Vc group.

### Effect of BCP on LDH Leakage of H9c2 Cells With OS Injury

**Figure 6:**
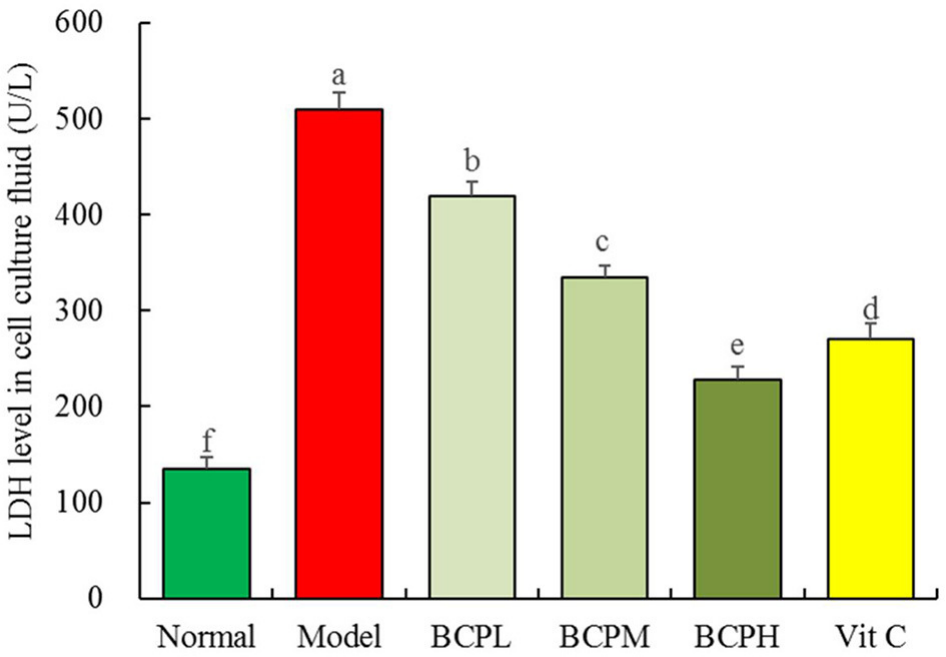
Effect of BCP on LDH level of H9c2 cells with OS injury. ^a−*f*^Mean values with different letters over the bar are significantly different (*P* < 0.05) according to Tukey's honestly significant difference.

The LDH leakage was the lowest in the normal group (135.67 ± 12.01 U/L) and the highest in the model group (508.93 ± 18.33 U/L) (Figure 6). BCP lowered the LDH leakage of OS-injured cell culture fluid, and the higher the BCP concentration, the lower the LDH leakage. The LDH leakage in the Vc group was lower than that attained by the same concentration of BCP.

### Effect of BCP on the Amounts of SOD, MDA, GSH, and CAT in H9c2 Cells With OS Injury

Compared with the normal group, the amounts of SOD, GSH, and CAT within the myocardial cells of the model group decreased, while MDA increased, with differences that were statistically significant (Table 6). Compared with the model group, the amounts of SOD, GSH, and CAT within the cells increased after being treated with low, medium, and high concentrations of BCP. The amount of MDA decreased, all with significant changes according to the increased BCP concentration, with differences that were statistically significant (*P* < 0.05). The regulatory effects of Vc were weaker than those of BCPH, but stronger than those of BCPH and BCPL.

**Table 6:** SOD, MDA, GSH, and CAT contents of H9c2 cells with OS injury.

**Group**	**SOD (U/gprot)**	**MDA (nmol/gprot)**	**GSH (μmol/mg)**	**CAT (U/gprot)**
Normal	184.32 ± 11.55^a^	0.88 ± 0.07^a^	69.81 ± 5.26^a^	145.27 ± 15.20^a^
Model	53.60 ± 4.71^e^	6.82 ± 0.34^d^	22.83 ± 3.71^e^	39.82 ± 4.27^e^
BCPL	79.36 ± 8.32^d^	5.12 ± 0.31^c^	31.82 ± 2.67^d^	59.67 ± 5.25^d^
BCPM	108.97 ± 6.77^c^	3.06 ± 0.29^c^	45.08 ± 4.33^c^	84.36 ± 7.99^c^
BCPH	142.57 ± 10.84^b^	1.87 ± 0.25^b^	56.34 ± 2.29^b^	112.46 ± 8.33^b^

a−e *Mean values with different letters over the same column are significantly different (P < 0.05) according to Tukey's honestly significant difference*.

### Effect of BCP on the mRNA Expression of Bcl-2, Bax, caspase-3, Nrf2, HO-1, and NQO1 in H9c2 Cells With OS Injury

**Figure 7:**
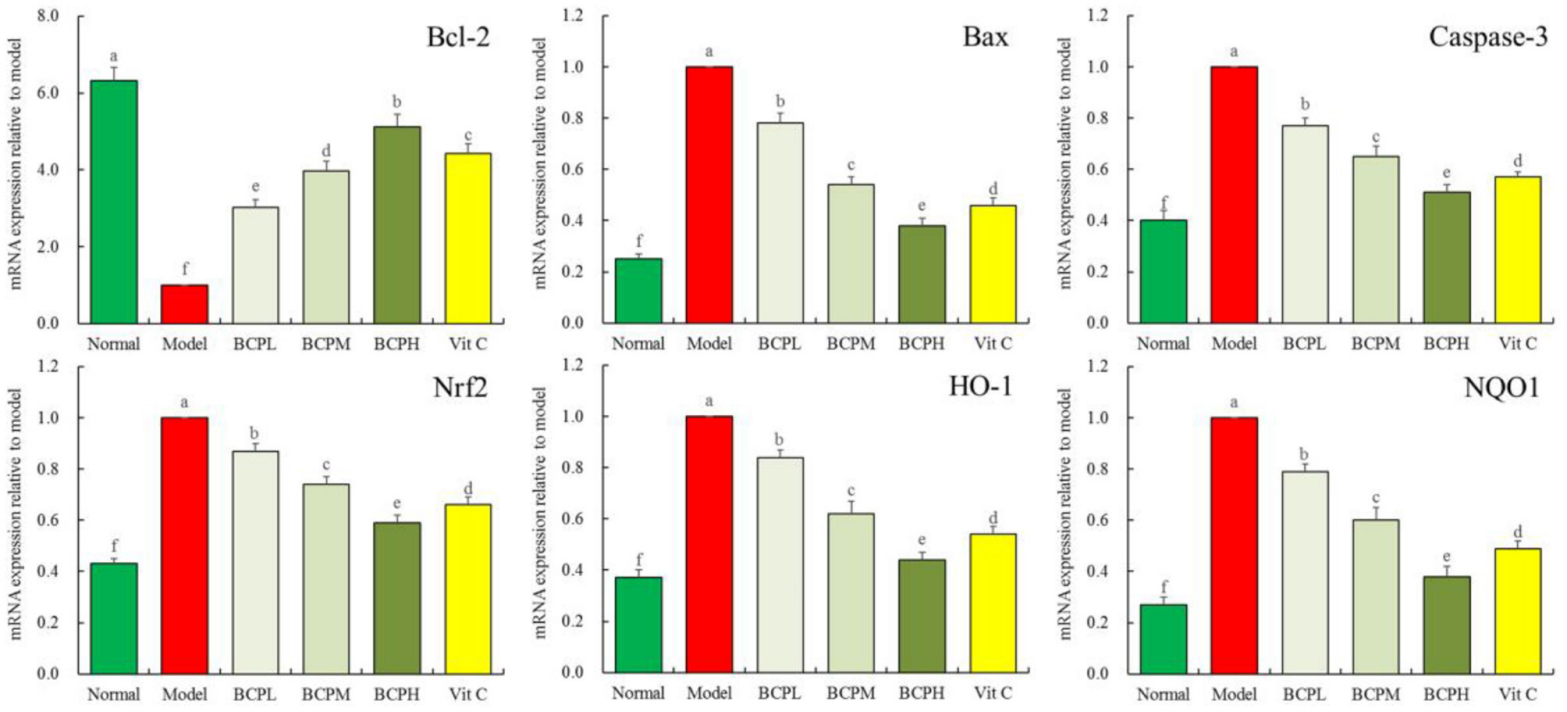
Effect of BCP on mRNA expressions of Bcl-2, Bax, Casepase-3, Nrf2, HO-1, and NQO1 of H9c2 cells with OS injury. ^a−*f*^Mean values with different letters over the bar are significantly different (*P* < 0.05) according to Tukey's honestly significant difference.

The qPCR analysis (Figure 7) indicated that compared with the normal groups, there was increased mRNA expression of Bax, caspase-3, Nrf2, HO-1, and NQO1 in myocardial cells in the model group, while Bcl-2 expression decreased, with differences that were all statistically significant (*P* < 0.05). Compared with the model group, after the BCP and Vc treatments, there was increased Bcl-2 expression within the myocardial cells, while the expression of Bax, caspase-3, Nrf2, HO-1, and NQO1 decreased. The change was greater with increasing BCP concentration, and the expression of BCPH was closest to that of the normal group.

### Effect of BCP on the mRNA Expression of Nrf2 in H9c2 Cell Nuclei With OS Injury

**Figure 8:**
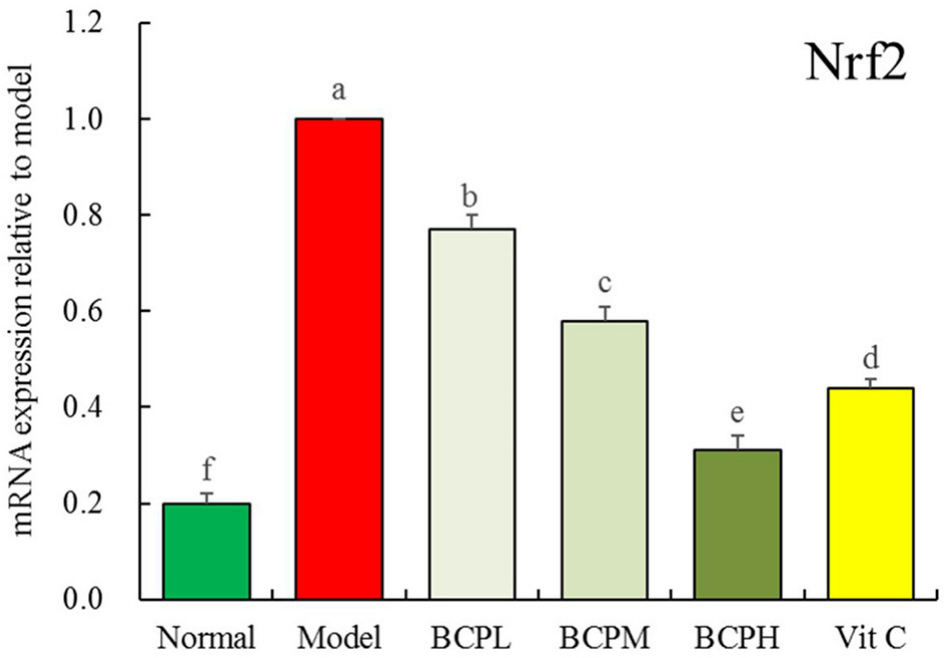
Effect of BCP on mRNA expressions of Nrf2 of H9c2 cell nucleus with OS injury. ^a−*f*^Mean values with different letters over the bar are significantly different (*P* < 0.05) according to Tukey's honestly significant difference.

The qPCR analysis (Figure 8) indicated that the mRNA expression of Nrf2 in the model group was also the strongest, and BCP reduced the expression with an effect that was stronger than that of Vc.

### Effect of BCP on the Protein Expression of Bcl-2, Bax, Caspase-3, Nrf2, HO-1, and Cyt C in H9c2 Cells With OS Injury

**Figure 9:**
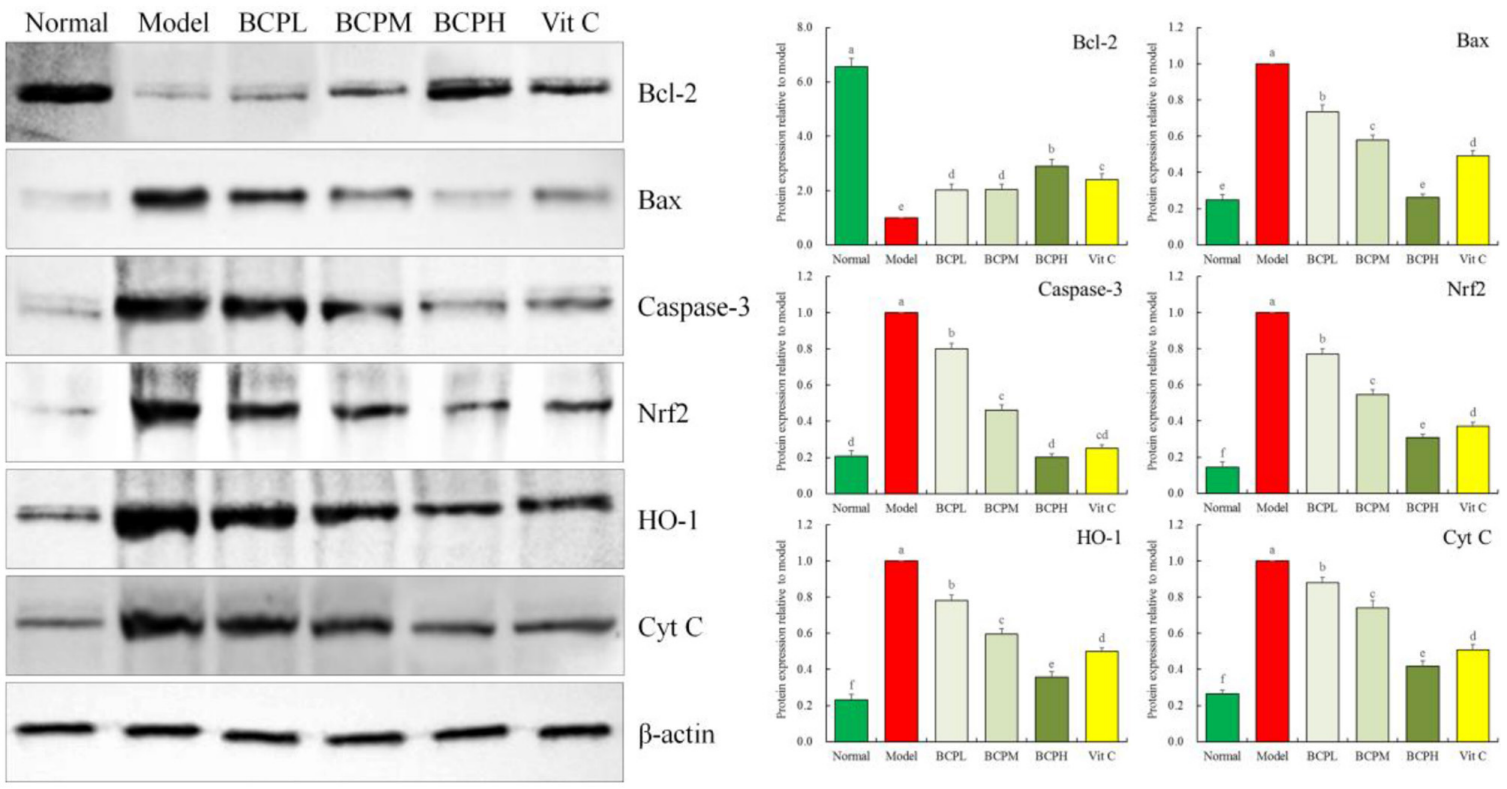
Effect of BCP on protein expressions of Bcl-2, Bax, Casepase-3, Nrf2, HO-1, and Cyt C of H9c2 cells with OS injury. ^a−*f*^Mean values with different letters over the bar are significantly different (*P* < 0.05) according to Tukey's honestly significant difference.

qPCR and western blot analysis (Figure 9) indicated that the trends for Bcl-2, Bax, caspase-3, Nrf2, and HO-1 protein expression in the experiment were the same as those observed for mRNA expression in the qPCR experiment. The protein expression of Cyt C in the normal group was the weakest, but was the strongest in the model group. BCP reduced the expression compared to the model group, and the expression of the BCPH group was the closest to that of the normal group. Vc also reduced Cyt C expression, and the Cyt C expression of the Vc group was only stronger than that of the normal and BCPH groups.

### Effect of BCP on the Mitochondrial Membrane Potential of H9c2 Cells With OS Injury

**Figure 10:**
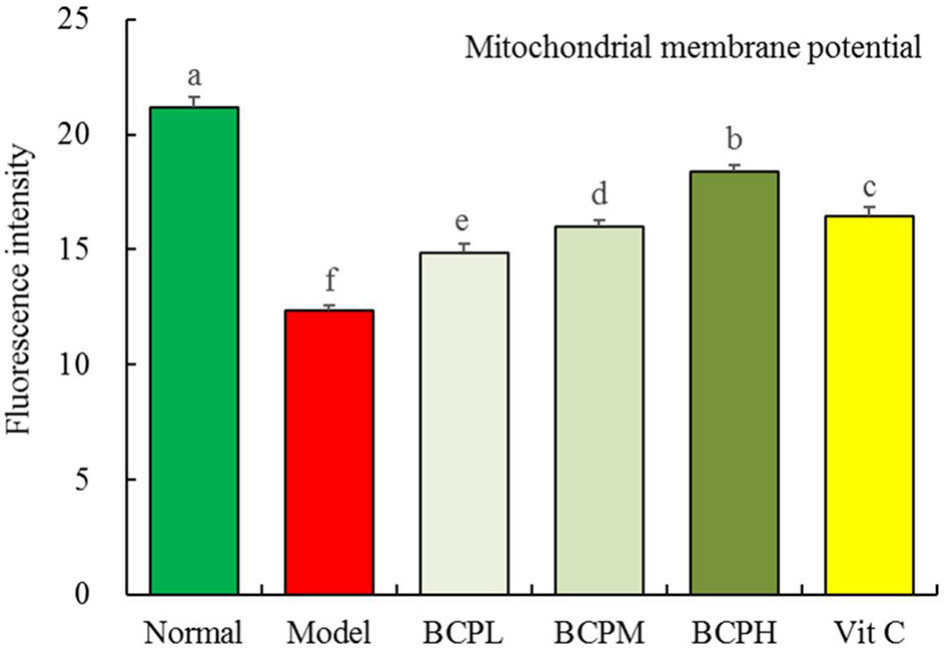
Effect of BCP on Mitochondrial membrane potential of H9c2 cells with OS injury. ^a−*f*^Mean values with different letters over the bar are significantly different (*P* < 0.05) according to Tukey's honestly significant difference.

Compared with the normal group, the mitochondrial membrane potential of cells treated with hydrogen peroxide decreased (Figure 10). BCP inhibited the decrease in the mitochondrial membrane potential caused by the oxidative damage due to hydrogen peroxide. The effect was enhanced with increasing concentration, with a stronger effect from BCP as compared to Vc at the same concentration.

## Discussion

Although the death rate for acute myocardial infarction decreases when early thrombolysis, percutaneous coronary intervention, or coronary artery bypass grafting are administered, acute myocardial infarction is still the leading cause of death and disability worldwide (24). After acute myocardial infarction, the patients will inevitably experience left ventricular reconstitution mediated by oxidative stress and myocardial cell apoptosis, and if oxidative stress and myocardial cell apoptosis are inhibited, this may reduce the extent of reconstitution (25). The essence of OS is the large generation of ROS that exceeds the scavenging ability of the body, resulting in injuries to the lysosomes and mitochondria (26). H_2_O_2_ is an oxidative metabolite *in vivo* that causes cell injury by strongly active oxygen radicals produced as H_2_O_2_ reacts with iron ions within the cell nucleus. H_2_O_2_ can also promote protein polymerization through the lipid oxidation metabolite MDA, which induces cell apoptosis (27). Our study also shows that H_2_O_2_ caused a decrease in the survival rate of H9c2 cells, and BCP can protect H9c2 cells as it increases the survival rate of H9c2 cells with oxidative injury caused by H_2_O_2_.

DPPH is a synthetic organic free radical that is commonly used to analyze antioxidant activity based on its ability to scavenge DPPH free radicals. The ABTS clearance rate is an important index for measuring antioxidant capacity. After oxidation, ABTS forms a relatively stable blue-green compound. After ABTS reacts with an antioxidant, the color of the solution is lightened. Therefore, the reduced absorbance of the reaction solution indicates the strength of its antioxidant effect (28). It has been proven that the stronger the reducing power of the active substance *in vitro*, the stronger the antioxidant capacity (29). In this study, BCP exhibited a strong antioxidant effect *in vitro*, as compared to Vc.

Under normal physiological status, the body contains relatively low amounts of serum LDH, and LDH within the cells is released in large amounts only after cell membrane injury occurs. As the major endogenous antioxidant *in vivo*, SOD removes excess oxygen radicals *in vivo* to reduce mitochondrial injury and maintain cell stability (30). In the BCP treatment group, the LDH content in the supernatant and MDA in cells significantly decreased, and the SOD in cells significantly increased, compared with that of the model group. This indicates that BCP has a satisfactory effect on antioxidant injury. GSH and CAT are also important antioxidants in the body, and they inhibit oxidative injury and protect the body *in vivo* when oxidative stress occurs (31, 32). The measurements of GSH and CAT also indicated that BCP can increase the antioxidant capability of cells. The antioxidation mechanism that occurs before, during, and after myocardial cell injury is not yet clear, although this remains the research emphasis of the current study. It is hoped that further understanding of the protection afforded by BCP will provide a new reference direction for future R&D of new medicines or functional foods that act against myocardial injury.

Apoptosis is known as programmed cell death, whose morphological changes are characterized by cell shrinkage, karyolysis, and DNA breakage (33). Myocardial cell apoptosis is characterized by mitochondrial apoptosis, in which the Bcl-2 family participates. The Bcl-2 family mainly consists of pro-apoptotic protein Bax and inhibitor of apoptosis protein Bcl-2, which can determine the degree of cell necrosis and apoptosis through regulating the permeability of the mitochondrial membrane (34). The caspase family also plays an important role in cell apoptosis. When Bax combines with the mitochondrial membrane, the ion concentration between the inner and outer membranes of the mitochondria changes, which causes Cyt C to flow into the cytoplasm, forming an apoptosome with caspase-9 containing cysteine and activating caspase-3 to induce cell apoptosis (35). Our qPCR and western blot experimental findings show that BCP increases Bcl-2 and reduces Bax and caspase-3 in terms of mRNA and protein expression quantity, which indicates that BCP protects the myocardial cells by inhibiting the mitochondrial apoptotic pathway.

As a regulatory factor of oxidative stress, Nrf2 inhibits the oxidative stress response, with its level regulated by HO-1 (36). Normally, Nrf2 exists in the cytoplasm as an inactive dimer with Keap1, and it can quickly separate from Keap1 and enter the cell nucleus after OS injury. In the nucleus, Nrf2 plays a protective role by enabling the expression that can encode antioxidant protein genes and activate the phase II antioxidant enzyme HO-1 at the downstream (37). At the same time, studies showed that Nrf2 also upregulates the expression of Bcl-2 and initiates anti-apoptosis (38). When Nrf2 induces its downstream antioxidant enzymes SOD, CAT, GSH, and HO-1, the body is protected from oxidative stress (39). Additionally, Nrf2 and the inhibitor Keap1 are present in the cytoplasm. After Keap1 is degraded, Nrf2 translocates to the nucleus and tends to bind to the promoter upstream of the HO-1 or NQO1 genes to induce their expression, thereby acting as an antioxidant (40). After oxidative stress, the expression of Nrf2 and its regulated HO-1 and NQO1 genes increases, and therefore, the high expression of these mRNAs and proteins appeared in the model group. After being subject to oxidative stress, cells were treated with BCP, the oxidative stress state was controlled, and the expression of the NRF/HO-1 pathway decreased, which normalized and stabilized subsequent expression and cell growth. The expression of Nrf2 may gradually resume so that it binds with Keap1, but then it will not express its antioxidant capabilities.

We found through qPCR and western blot experiments that BCP can play an antioxidation role by regulating the expression of nucleus transfer of Nrf2 and HO-1 at the mRNA and protein levels, indicating that the antioxidation action of BCP is possibly related to the Nrf2/HO-1 signaling pathway. However, which upstream gene transcriptions activate Nrf2 for cell nuclei entry and which cell factors promote it to activate downstream HO-1 are yet to be researched.

Mammalian mitochondria play an important role in apoptosis, and Cyt C is an apoptosis-related protein present on the inner mitochondrial membrane. The occurrence of apoptosis is associated with the release of Cyt C from within mitochondria into the cytosol. Studies have shown that decreased mitochondrial transmembrane potential is a hallmark of the occurrence of apoptosis, and the maintenance of mitochondrial membrane potential is associated with the degree of opening of the mitochondrial membrane permeability transport pore. Normally, the mitochondrial membrane potential regulates the exchange of substances between the inner and outer mitochondria, and when stimulated by DNA damaging agents or oxidative stress, the mitochondrial membrane permeability transport pore opens, and the mitochondrial membrane potential decreases so that the Cyt C originally existing in the mitochondria enters into the cytosol, triggering the apoptotic cascade (41). In the current study, BCP equally regulates the mitochondrial membrane potential and Cyt C expression, thus protecting cells from damage and apoptosis triggered by oxidative stress, with a stronger effect than that of Vc.

In medicine, mannitol is used as an effective diuretic that can decrease intracranial pressure and intra-ocular pressure, and thus, it is used as a kidney treatment. It is also used as a sugar substitute, and one industrial production method involves extracting it from sea products such as kelp (42). Ribose is one of the components of RNA, as well as a material that molecules such as ATP and NADH require for biochemical metabolism, which is essential to the body. Glucosum can be used as a nutritional supplement in the form of an oral liquid or intravenous injection, and it can also be used as a reducing agent to prevent oxidation in medicine (43). Galactose, which exists in the brain and nerve tissue, is the cerebroside component that constitutes the cerebral nervous system, and it is an important component of some glycoproteins (44). Xylose serves to link cells for conjugation to receptors on the cell membrane, with anti-bacterial, anti-fungal, and intestinal probiotic growing functions (45). In the current study, BCP, which is composed of these functional monosaccharides, exhibits excellent antioxidant ability that can protect cells from the effects of oxidative stress. Component analysis shows that these effects are due to a new chemical structure formed by 5 monosaccharides in BCP. However, the current study was limited to cell experiments *in vitro*, and further experiments are required *in vivo* to further clarify the regulatory mechanism of BCP on the pathways *in vivo*.

In conclusion, BCP exerted a protective effect on OS-injured H9c2 cells, and its mechanism of action may be related to the Nrf2/HO-1 signaling pathway upregulated by BCP through 5 monosaccharides that constitute its structure, which resulted in an increase in the activity of the antioxidant enzymes SOD, GSH, and CAT in myocardial cells to inhibit cell apoptosis. Further studies on the molecular mechanism of BCP are required to clarify its targets of action, which will provide a theoretical basis for the continuous development of BCP.

## Data Availability Statement

The original contributions presented in the study are included in the article/supplementary material, further inquiries can be directed to the corresponding author/s.

## Author Contributions

YJ performed the majority of the experiments and wrote the manuscript. WZ, XZ, and YW contributed to the data analysis. DY and SL designed, supervised the study, and checked the final manuscript. All authors contributed to the article and approved the submitted version.

## Conflict of Interest

The authors declare that the research was conducted in the absence of any commercial or financial relationships that could be construed as a potential conflict of interest.
